# Assessing training needs in infectious disease management at major ports, airports and ground-crossings in Europe

**DOI:** 10.1186/s12889-021-11008-z

**Published:** 2021-05-29

**Authors:** Doret de Rooij, Evelien Belfroid, Christos Hadjichristodoulou, Varvara A. Mouchtouri, Jörg Raab, Aura Timen, for the EU HEALTHY GATEWAYS Joint Action consortium

**Affiliations:** 1 Centre for Infectious Disease Control, National Institute for Public Health and the Environment, Bilthoven, The Netherlands; 2 Athena Institute, Free University, Amsterdam, The Netherlands; 3 Department of Hygiene and Epidemiology, University of Thessaly, Thessaly, Greece; 4 Department of Organization Studies, School of Social and Behavioral Sciences, Tilburg University, Tilburg, The Netherlands

**Keywords:** Points of entry, Port, Airport, Ground-crossing, Infectious disease control, Training, Exercise, Education, Training needs assessment

## Abstract

**Background:**

The implementation of core capacities as stated in the International Health Regulations (IHR) is far from complete, and, as the COVID-19 pandemic shows, the spreading of infectious diseases through points of entry (POEs) is a serious problem. To guide training and exercises, we performed a training needs assessment on infectious disease management among professionals at European POE.

**Methods:**

We disseminated a digital questionnaire to representatives of designated airports, ports, and ground-crossings in Europe. Topics were derived from the IHR core capacities for POEs. Based on the importance (4-point Likert scale) and training needs (4-point Likert scale), we identified the topics with the highest priority for training. These results were put in further perspective using prior experience (training < 3 year, exercise < 5 years, events < 5 years). Also, preferences for training methodologies were assessed.

**Results:**

Fifty questionnaires were included in the analyses, representing 50 POEs from 19 European countries. Importance is high for 26/30 topics, although scores widely vary among respondents. Topics with a high training need (16/30) are amongst others the handling of ill travelers; using and composing the public health emergency contingency plan, and public health measures. Respondents from ports and airports attribute equal importance to most topics, but respondents from ports showed higher training needs on 75% of the topics. POEs are unevenly and generally little experienced. The most preferred training methods were presentations. Simulation is the preferred methodology for training the handling of ill or exposed travelers.

**Conclusions:**

The European workforce at designated ports, airports and ground-crossings has a different level of experience and perceives varying importance of the topics assessed in our study. We identified the topics on which training is required. We call for European collaboration between POEs to agree upon the importance of infectious disease management, and to jointly build a trained and prepared workforce that is ready to face the next crisis.

**Supplementary Information:**

The online version contains supplementary material available at 10.1186/s12889-021-11008-z.

## Background

In the globalized world, infectious diseases spread easily from one country to another via travelers and goods [1–5]. The intercontinental spread of SARS (2003), A/H1N1v (2009), Ebola Virus Disease (2014–2015) and COVID-19 (2019–20) are major examples from recent history. These examples show that threats easily spread through air-, maritime- and land travel and that countries need to be prepared to respond to outbreaks but also to prevent them through measures at their points of entry. To safeguard a collaborative effort in preventing the cross-border spreading of disease, the International Health Regulations (IHR) are being abided globally, and the Decision 1082 of the European Commission in Europe [6, 7]. According to IHR, countries are committed to designate at least one airport and one port, and may designated a ground-crossing, where the core capacities should be in place to respond effectively to infectious disease threats [6].

It is hard to say how prepared Europe’s designated ports, airports and ground-crossings – together called points of entry (POEs) – are to respond to infectious disease threats, but there are serious indications that they are not prepared enough yet. The IHR yearly self-assessment in 2019 showed that less than 60% of the core capacities have been implemented in Europe [8, 9]. And this is not even the full equation since many countries do not report their preparedness status at all, and those who do, have self-assessed their status. The World Health Organization (WHO) Joint External Evaluations (JEE) (2016–2018), which until now have been performed in only 14 of 49 countries in the WHO EURO region, show a wide variety of capacity implementation among countries both in daily situations as well as for a public health emergency of international concern (PHEIC) [10]. Worryingly, other studies show a general lack of sustainable training programs and a general lack of awareness of infectious disease preparedness among POE professionals [11, 12].

Because of the large number of continental flights and the way travel restrictions among European countries have decreased over the past decades, European POEs are largely dealing with the same body of travelers. Their interdependency urges a joint level of preparedness at European POEs, as is currently supported by the European Union (EU) Joint Action Healthy Gateways (2018–2021) [13]. However, complete contact networks among POEs are lacking and a study among the European workforce as a whole has not been performed. In this way, it is unclear, from a workforce perspective, which issues have the highest priority and how training should be performed. For example, training needs could vary from performing a risk assessment, the implementation of measures, or routine inspections. And training designs might differ between a series of lectures, interactive discussion of case studies, or a full-scale simulation exercise.

Therefore, in this study, we reached out to Europe’s designated ports, airports and ground-crossings and performed the first European wide training needs assessment from a POE perspective. To guide future training efforts, we aimed to identify training priorities, and corresponding training methods.

## Methods

This study was conducted between August 2018 and August 2019. Digital questionnaires for airports, ports and ground-crossings were developed seperately and disseminated to designated ports, airports and ground-crossings in Europe with the aim to assess training needs from a workforce perspective. We collected data on the importance of different topics, the training needs for these topics, and prior, experience with infectious disease management (preparedness and response) at POEs.

### Study population

We invited professionals involved in infectious disease preparedness and response at European designated POEs to complete our questionnaire. Because no validated and complete list of European designated POEs exists, we used an indirect sampling method. First, the questionnaire was disseminated via a link in an email by the coordinator of the EU Joint Action Healthy Gateways to the national representatives of all 26 participating countries in this EU Joint Action. Then, these national partners were asked to forward the link to a selected professional per designated POE who was involved in infectious disease preparedness and response and could represent this designated POE regarding their training needs. A single respondent represented a POE but was encouraged to consult colleagues during completion.

### The questionnaire

The digital questionnaire was built in Formdesk [14]. We used the IHR Annex 1B ‘core capacity requirements for designated airports, ports and ground crossings' to extract the different issues for POEs (topic A – H) which were further divided into corresponding subtopics (A1 – H3) [6]. In line with IHR Annex 1B, we made a distinction between topics that are required ‘at all times’ (topics A, B, C, D) and those that are required during a PHEIC (topics E, F, G, H). For airports and ground-crossings, 29 subtopics were extracted. The questionnaire for ports had one extra subtopic on ballast water management (subtopic C5). Terms specific for the type of POE were adjusted accordingly (e.g. aircraft; ship; vessel). This resulted in similar questionnaires for ports, airports and ground-crossings, as shown in Additional file 1, Additional file 2, and Additional file 3 respectively. The questionnaire was pilot-tested by two professionals involved with hygiene and infectious disease management at POEs. Based on their feedback, we made textual changes and included the option to consult colleagues during the completion of the questionnaire.

The first part of the questionnaires captured demographic characteristics, such as their country of origin, the designated PoE, gender, job title, involvement in managerial and/or operational tasks, working level (local, regional, national), sector (private, public), and years of working experience in the current job. Then, respondents scored importance and training needs using 4-point Likert scales (3 = high, 2 = moderate, 1 = low, 0 = no, or I don’t know). Lastly, respondents rated per topic their previous education or training in the last 3 years (yes, no, I don’t know), and their training methods of preference (‘presentations’, ‘case-studies’, ‘discussions’, ‘e-modules’, ‘simulations’, ‘no preference’, or ‘other, namely … ’; multiple answers were allowed). Respondents were invited to fill out their e-mail address for follow-up questions. All other questions in the questionnaire were mandatory. The questionnaires were distributed between 23 October and 19 November 2018, and 7 February and 10 March 2019. Several reminders were sent.

#### Additional data collection

We sent a questionnaire to respondents that had provided their e-mail address for follow up questions on training and practical experience in the past 5 years (Additional file 4). We asked whether and how often multi-disciplinary exercises were scheduled and how often they had managed an event at their point of entry in the last year (2018) and in the last 5 years.

### Data preparation

Data were imported in Microsoft Excel and verified for correct storage and missing data, before analysis in IBM SPSS Statistics 24_0_0_1 (IBM Corp., Armonk, NY, USA) [15]. Questionnaires without any data other then demographic characteristics of the respondent were excluded, as were double submissions (the first submission was kept) or those not agreeing with the privacy statement. We calculated the response rates for countries. A non-response analysis (one sample t-test) on country-level was performed using capacity scores from the IHR self-assessment of 2018 [9]. For the follow-up questionnaire, a non-response analysis was performed based on working level and average training needs (one sample t-test). We translated nominal variables into binary variables for each answer option. Experience with real events was dichotomized into any experience (value = 1) vs. no experience (value = 0) in order to analyze training needs among these groups. We analysed the consistency of importance and training needs and developed constructs of scores per main topic if Cronbach’s alpha > 0,7 [16].

### Data analysis

Baseline characteristics were calculated. The previous experience, importance, training needs, and preference for methodologies were analysed primarily by calculating the modus. Regarding importance and training needs, the frequencies on ‘low’ and ‘no’ were added up and indicated as ‘low/no’. Also means and standard deviations were calculated to enable further analyses. To assess to what extent importance and training need were valued differently, statistical differences between importance and training needs were analysed using the Wilcoxon-signed rank test. The same analyses were performed for all POEs together and for ports, airports and ground-crossings separately.

Furthermore, the relations between experience from prior training and importance, and experience from prior training and training needs were assessed using a Spearman rank-order correlation. A t-test was performed to compare the experience with real cases (any real cases < 5 year; none) and training needs. A limit of α ≤ .05 was used for statistical significance.

## Results

### Respondent characteristics

The questionnaire was completed 58 times, representing POEs in nineteen countries. Of these 58 questionnaires, eight questionnaires were excluded from the main analyses because they were a double submission on the same working level (*n* = 4), or from a non-designated POE (*n* = 4). The port of Finland was non-designated, but its representatives’ results could be included after confirmation that designation is in process and the port already functions as such. This led to a response rate on country level of 73%. The non-response analysis showed that non-responding countries score slightly lower on the IHR core capacities for PoE than countries that completed the questionnaire (mean 53.3% vs. 55.6% respectively; *p* = <.001). Since sampling was performed indirectly, a wide variety of professionals responded to the questionnaire. Fourteen respondents described their job as environmental or health inspector; eight as an environmental, health or public health officer; six as a chief; head or director position; seven as a medical doctor; and several others had single job titles. Further characteristics of the 50 included respondents are shown in Table 1. The second questionnaire to collect additional data was filled out by thirteen of the 50 respondents (26%). A non-response analysis for this set did not show signficiant difference (responders’ Training Needs = 2.07 vs. non-responders’ Training Needs = 1.83; *p* = .320).

**Table 1:** Baseline characteristics of respondents for all POEs together and per POE type

Variable	All POE (n, (%))	Ports (n, (%))	Airports (n, (%))	Ground-crossings (n, (%))
**Number (n)**	50 (100)	27 (54.0)*	18 (36)*	5 (10)*
**Countries**	19 (100)	17 (89)*	17 (89)*	4 (21)*
**Response Rate for countries**	19/26 (73)	17/26 (65)	17/26 (65)	4/26 (15)
**Gender**				
Male	26 (52)	14 (52)	9 (50)	3 (60)
Female	24 (48)	13 (48)	9 (50)	2 (40)
**Working level**				
National level	20 (40)	7 (26)	10 (56)	3 (60)
Regional level	16 (32)	10 (37)	4 (22)	2 (40)
Port level	14 (28)	10 (37)	4 (22)	0 (0)
**Sector**				
Public sector	48 (95)	26 (96)	17 (94)	5 (100)
Private sector	2 (5)	1 (4)	1 (6)	0 (0)
**Highest completed education**				
Secondary education	2 (4)	1 (4)	0 (0)	1 (20)
Vocational education	1 (2)	1 (4)	0 (0)	0 (0)
Higher professional education	5 (10)	3 (11)	2 (11)	0 (0)
University	42 (84)	22 (81)	16 (89)	4 (80)
**Years experience in the current job**	14.8 (8.2)^	14.9 (7.7)^	14.5 (9.6)^	15.6 (7.2)^

*)% of All PoE; ^Mean (Standard Deviation); *POE* point of entry, *n* number

### Experience

Respondents have generally little experience with prior education or training, multi-disciplinary exercises and real events. Education and training received in the last 3 years ranged between 8 and 44% of respondents (Table 2). Lowest percentages are identified for *H. Affected animals* and *F. Recommended measures* in case of a PHEIC; highest for topic *C. routine inspections* and *topic A. Different health risks*. Experience from prior training or education did weakly correlate (coeff. <.31) with importance and training needs.

**Table 2:** Results of importance and training needs per topic for all respondents (ports, airports and groundcrossings)

Topics	Training in last 3 years	Importance		Training needs		Difference	High score
	***n (%)***	***Modus (H; M; L/N)***	***mean (SD) (0 = N; 1 = L; 2 = M; 3 = H)***	***Modus (H; M; L/N)***	***mean (SD) (0 = N; 1 = L; 2 = M; 3 = H)***	***∆ (Z-score (p-value))***^c^	
A – Knowledge of public health risks	20 (40)	M	2.09 (.70) (α.842)^b^	M	1.81 (.69) (α.857)^b^	−1.885 (.059)	
A1 – biological agents		H	2.44 (.76)	M	1.94 (.79)	−3.570 (<.001)^a^	
A2 – chemical agents		M	2.00 (.80)	M	1.81 (.79)	−1.153 (.249)	
A3 - radiological agents		M	1.77 (.83)	M	1.66 (.82)	−.590 (.555)	
B – Safe environment	15 (30)	H	2.16 (.72) (α.859)^b^	L/N	1.75 (.86) (α.911)^b^	−3.847 (<.001)^a^	
B1 - Inspection programs		H	2.39 (.81)	H; M	1.94 (1.00)	−3.598 (<.001)^a^	!
B2 - Vector control at/near the PoE		H	2.27 (.93)	H; M	1.98 (.98)	−2.083 (.037)^a^	!
B3 - Food- and water safety		H	2.38 (.92)	L/N	1.84 (1.06)	−3.842 (<.001)^a^	
B4 - Public washrooms; waste management		M	2.06 (.87)	L/N	1.54 (1.03)	−3.701 (<.001)^a^	
B5 - Air quality		L/N	1.71 (1.01)	L/N	1.46 (.98)	−1.989 (.047)^a^	
C – Routine vessel inspections	22 (44)	H	2.23 (.79) (α.926)^b^	H; L/N	1.87 (.92) (α.971)^b^	−3.178 (.001)^a^	!
C1 – standard operating procedures;		H	2.47 (.79)	H	2.04 (.99)	−3.631 (<.001)^a^	!
C2 – Sewage; solid- and medical wastes;		H	2.06 (.99)	L/N	1.73 (.97)	−2.724 (.006)^a^	
C3 –Food- and water safety;		H	2.29 (.96)	H; L/N	1.88 (1.05)	−3.021 (.003)^a^	!
C4 – Assessment of required health documents;		H	2.31 (.96)	H	1.90 (1.10)	−2.634 (.008)^a^	!
C5 – Ballast water management^a^.		H; L/N	1.92 (1.10)	H	1.92 (1.02)	−.104 (.917)	!
D – Ill travelers	16 (32)	H	2.41 (.86) (α.952)^b^	H	2.15 (.90) (α.948)^b^	−2.374 (.018)^a^	!
D1 – Use of protective equipment;		H	2.50 (.85)	H	2.24 (.95)	−2.368 (.018)^a^	!
D2 – Safe removal of travellers for assessment, care, quarantine or isolation;		H	2.42 (.90)	H	2.20 (.96)	−1.969 (.049)^a^	!
D3 – Triage;		H	2.44 (.97)	H	2.17 (1.02)	−2.156 (.031)^a^	!
D4 – Approaching diagnostic facilities and medical services.		H	2.29 (.94)	H	2.00 (.97)	−2.442 (.015)^a^	!
E – PHEC plan	17 (34)	H	2.49 (.72) (α.836)^b^	M	1.92 (.93) (α.942)^b^	−4.001 (<.001)^a^	
E1 – Composing and updating;		H	2.56 (.76)	M	1.94 (1.00)	−3.665 (<.001)^a^	
E2 – Adequate and timely usage;		H	2.48 (.79)	M	2.02 (.98)	−3.535 (<.001)^a^	
E3 – Arrangements with local medical services.		H	2.43 (.89)	M	1.87 (1.00)	−3.945 (<.001)^a^	
F – Prevention measures	10 (20)	H	2.38 (.66) (α.836)^b^	H	2.08 (.88) (α.908)^b^	−2.560 (.010)^a^	!
F1 – disinsection, deratting, disinfection, decontamination;		H	2.60 (.64)	H	2.17 (.91)	−3.377 (.001)^a^	!
F2 – Treating goods, baggage, cargo or postal parcels;		H	2.23 (.88)	H	2.00 (1.03)	−2.000 (.046)^a^	!
F3 – Treating containers or vessels.		H	2.31 (.75)	M	2.08 (.92)	−1.932 (.053)	
G – Ill and exposed travelers	15 (30)	H	2.53 (.68) (α.923)^b^	H	2.13 (.87) (α.939)^b^	−3.432 (.001)^a^	!
G1 – Use of protective equipment;		H	2.67 (.66)	H	2.28 (.91)	−3.260 (.001)^a^	!
G2 – Use of space for assessment, care, quarantine or isolation;		H	2.55 (.74)	H	2.17 (.90)	−3.378 (.001)^a^	!
G3 – Interview and triage;		H	2.50 (.80)	H	2.04 (1.01)	−3.359 (.001)^a^	!
G4 – Safe transfer of suspected travelers		H	2.42 (.82)	H/M	2.02 (.95)	−3.285 (.001)^a^	!
H – Affected animals	4 (8)	L/N	1.71 (1.04) (α.950)^b^	L/N	1.41 (1.08) (α.979)^b^	−2.534 (.011)^a^	
H1 – Arrangements with local veterinary services;		L/N	1.72 (1.07)	L/N	1.45 (1.06)	−1.880 (.060)	
H2 – Infection control;		H; L/N	1.82 (1.13)	L/N	1.48 (1.13)	−2.348 (.019)^a^	
H3 – Care or treatment		L/N	1.62 (1.07)	L/N	1.31 (1.12)	−1.949 (.051)	

^a^Only applicable for ports; ^b^ Crohnback’s alpha; ^c^ Wilcoxon-signed rank test. *H* high, *M* moderate, *L* low, *N* no, *!* a topic with high importance and high training need

Experience with real events ranged between 0 and 20 events in the last 5 years, with a median of 1. Multi-disciplinary exercises were regularly performed by five of 13 POEs, with a range between 0 and 10 and a median of 1. Real events and multidisciplinary exercises were equally divided between airports and ports; results for ground-crossings were insufficient. Respondents with experience with participating in exercises or real events showed higher training needs, although results are not significant (*p* = .229;.685 resp.).

### Importance

Twenty-four of the 30 topics scored high on importance, as shown in Table 2. Among these topics, the highest mean scores were identified for the use of personal protective equipment both in routine (D2) and response situations (G2), composing and updating the Public health emergency contingency (PHEC) plan (E1), hygienic public health measures (F1), and the handling of ill travelers (G1–3). Incongruent scores among respondents were identified for for ballast water management (C5) and infection control on animals (H2), with an equal frequency for high and low/no importance.

### Training needs

Sixteen of the 30 topics were scored high on training needs. Among these, the handling of ill travelers routinely (D) and during a PHEIC (G), preventive measures (F), Standard operating procedures (C1), and adequate and timely usage of the PHEC plan (E2) had the hightest mean score. Food- and water safety (B3, C3) and required health documents (C4) had an equally high frequency for high and low/no scores. All topics with a high training need, based on the modus, are also regarded highly important, as indicated with the ‘!’ in Table 2.

The scores on importance and training needs significantly differed for all topics except for six, being: chemical agents (A2), radiological agents (A3), ballast water management (C5), treating containers or vessels (F3), arrangements with local veterinary services (H1), and care or treatment of animals (H3). Of these topics, ballast water management (C5) was only assessed by ports and its analysis is based on a lower number of values. Of these topics, A2, A3, H1, H3 are among the lowest scored topics and regard other aspects than infectious disease control focused on humans.

The full dataset of rough and prepared data can be found in Additional file 5.

### Ports, airports, and ground-crossings

Generally, respondents from ports and airports reported higher importance and had higher training needs than respondents from ground-crossings. Due to the low response rate for ground-crossings, we are not able to provide results for ground-crossings specifically. Specific results for airports and ports are shown in Table 3. Difference among points of entry for different subtopics are shown in Additional file 6.

**Table 3:** Importance and training needs for ports and airports

Topic	Ports (***n*** = 27)			Airports (***n*** = 18)		
	Importance (mean (SD) – modus (n))	Training needs (mean (SD) – modus (n))		Importance (mean (SD) – modus (n))	Training needs (mean (SD) – modus (n))	
A –public health risks	2.02 (.83)a	1.78 (.77) Mod (16)		2.17 (.49) Mod (13)	1.82 (.62) Mod (13)	
B – Safe environment	2.38 (.70) High (16)	1.94 (.91) Mod (12)		1.96 (.69) Mod (9)	1.54 (.76) Mod (9)	
C – Routine vessel inspections	2.33 (.84) High (15)	2.03 (1.00) High (12)	!	2.10 (.77) High (8)	1.66 (.77) Mod (9)	
D – Ill travelers	2.38 (.90) High (15)	2.19 (.95) High (14)	!	2.60 (.70) High (12)	2.24 (.79) Mod (9)	
E – PHEC plan	2.60 (.67) High (18)	1.96 (1.00) Mod (10)		2.57 (.56) High (11)	1.94 (.72) High (12)	!
F – Prevention measures	2.44 (.70) High (14)	2.19 (.90) High (12)	!	2.41 (.57) High (8)	2.08 (.82) Mod (9)	
G – Ill and exposed travelers	2.58 (.67) High (16)	2.17 (.97) High (13)	!	2.61 (.64) High (12)	2.15 (.76) Mod (9)	
H – Affected animals	1.78 (1.02) Low/No (9)	1.56 (1.11) Low/No (11)		1.84 (.1.05) High(6);Mod (6)	1.46 (1.07) Low/No (8)	

*N* number, *SD* standard Deviation, *mod* moderate; ^a^equal scores for high, moderate and low/no;*!* a topic with high importance and high training need

Respondents from ports considered six out of eight topics highly important (B, C, D, E, F, G). Ports expressed high training needs for routine vessel inspections (C), the handling of ill travelers (D), prevention measures (F) and ill travelers during PHEIC situations (G). Highest discongurence among respondents for ports was identified for training needs regarding handling of affected animals (H). The discongruence is shown by the modus being low/no importance (*n* = 11), but also six respondents checked ‘I don’t know’ for this topic, and six scored it as having a high training need.

Airports also considered six of the eight topics highly important. A safe environment (B) and public health risks (A) were considered moderately important. Respondents from airports had a high training need for the PHEC plan (E). For all others topics, a moderate training need is identified.

### Preference for training methodologies

The training methodology of overall preference was the use of presentations (22%), followed by discussions (19%) and e-modules (19%). Simulation exercises were most preferred for training handling of ill travelers rountinely (21%) and during a PHEIC (20%). Respondents selected on average 2.56 prefered methodologies per topic. Least preference for a methodology was identified for affected animals (H) for which 14 respondents selected no methodology of preference. In the open answers, suggestions for other methodologies were made by four respondents. They suggested for different topics practical training for the use of personal protective equipment (for topic D and G), face-to-face training (for all topics), simulation MARSEC (for topic D), and on-site training (for topic A and B). Detailed results on training methodology preference can be found in Additional file 7.

## Discussion

In this study, we performed the first European wide training needs assessment from a POE perspective. This training needs assessment aimed to gain insight into the training needs on infectious disease management among dedicated staff at designated airports, ports, and ground-crossings in Europe. Handling ill travelers, public health measures at PoE, and routine inspections have the highest priority for training among ports, airports and ground-crossings together. Combining the moderate to high training needs, the low percentage of respondents that received recent training, and the few real events that were experienced, we call for additional training efforts to enhance the workforce preparedness at European POEs.

### Interpretation of the results

Our results are univocal regarding issues that are both important and have high training needs, such as the handling of ill travelers and several public health measures. Here, according to our sample, additional training efforts should be made. However, less clear is the conclusion for issues that are considered little or not important, such as handling animals, or health risks from a chemical or radiological essence. History can mark several chemical events affecting international travel and trade that require effective response by points of entry [17, 18]. Also, several cross-border public health events can be pointed outin recent history involving zoonoses and the transport of animals [19], such as tularemia [20], bovine spongiform encephalopathy or BSE [21], or avian influenza [22]. It is therefore of no surprise that these are stated in the core capacity list in the IHR and recent landmark guidelines.

Several interpretations are possible with regard to the fact that these animal and chemical threats seem to receive little attention. First, they might be scored as less important relative to the other topics instead of not being important at all in an absolute sense. Or second, respondents may be unaware of these issues being important or do not consider it a POE problem. The little experience from prior training, exercise and real cases that we identified prudently supports this second interpretation. Because if the workforce is not trained to focus on an issue, and no direct consequences follow from a lack of attention, one can concede to attribute little importance to it. Chemical events have shown to be disruptive. And as the number of zoonotic (re) emerging diseases increases [19, 23], from a one health perspective animal handling does indeed require attention in the training of POE personnel.

Also, the diverging training needs among respondents needs further attention. For many topics, we saw high training needs as well as low training needs. This incongruence indicates varying perspectives on infectious disease preparedness among POEs, which is not explained by differentiating between ports, airports and ground-crossings. Again, this is in line with the generally low and widely varying level of preparedness among POEs, as shown by the results of our study, the IHR self-assessment [9], and the Joint External Evaluations of IHR core capacities. In the light of increasing travel with and within Europe [24] and the experiences of the current COVID-19 pandemic, it is of utmost importance that the awareness for the role POEs in cross-border disease preparedness and control, and subsequently, the development of a prepared workforce is being implemented.

We identified a very low number of infectious disease events at POEs, with few exceptions reporting several events a year. This finding correlates with the results of a literature review conducted in 2013 in which less than 70 events were identified between 1990 and 2013 in the categories ‘European ports’, ‘the Mediterranean Sea’, or ‘worldwide’ [25]. The latter is named here because European crew and ships might be involved. The combination of the workforce’s little experience with events in practice, and the little attention of the topic in education, training and exercises in the last 5 years raises the question to what extent the training needs merely indicate a gut feeling, or are a reliable estimation related to real practice. Also, the low and highly varying number of events may suggest uneven chances for events to occur at different points of entry. Another possibility is that it signals an incomplete identification and notification of infectious disease at several POEs. What the right interpretation is needs to be studied by determining the differences in risk for events to occur at different per points of entry.

Airports, ports and ground-crossings perceive slightly different needs. Airports and ports have the highest training needs in PHEIC situations. This difference can be explained by several events. First, the large Ebola outbreak of 2014–2015 led to enhanced screening at ports and airports worldwide [26, 27]. In addition, in the meantime, major EU Joint Actions AIRSAN and SHIPSAN supported countries extensively with the development of effective infectious disease control at ports and airports [28, 29]. Ground-crossings, however, have received less attention since they only had a minor role in the spreading of Ebola and have not had a EU Joint Action aimed at enhancing their preparedness. However, a recent report shows a substantial and growing number of travelers that enter Europe via train and roadways and a suboptimal prepared workforce for dealing with infectious disease threats [30]. In combination with the current COVID-19 pandemic, we expect more attention for and awareness at ground-crossings on infectious disease management shortly.

The high preference for presentations as training methodology is hard to resolve with currently leading educational theories, such as the Adult Learning Theory. This theory promotes interactive, problem-based learning in real environments to be most effective [31]. Future organizers of training programs should note this discrepancy in preferences between our respondents and leading theories, and consider consulting didactic professionals during training development. Even more, because the literature on training in infectious disease control mostly leaves us here, as is shown in a recent but still unpublished literature review on effective training methods performed by this study’s authors. However, very promising tools have been developed and tested to enhance active learning and interaction during presentations, such as the use of audience response systems [32] and online methodologies. (Online) E-modules are the other preferred methodology and already better suited for problem-based learning and limited interaction among learners. Since there is a need for European-wide training at POEs, this might be a very suitable method to reach this geographically spread target group. Locally at POEs, however, our respondents simulation exercises for practical skills such as the use of personal protective equipment and the handling of ill persons.

### The Covid-19 pandemic

Between the data collection and reporting of this study, the COVID-19 pandemic has confronted many European POEs with the response to infectious disease threats on an unprecedented scale. News reports, first scientific publications and the authors’ experiences indicate that indeed several POEs perceived enormous challenges to handle cruise ships with cases on board [33, 34], implement public health measures at airports and on land-borders [35]. These events again emphasize, how important the capacity of personnel and organization and the necessary training at PoEs are. The results of this study outline the starting point from which, in January and February 2020, Europe’s POEs started the required ad hoc preparations for the COVID-19 crisis. This crisis, however, inevitable has lead to new insights on what kind of training is required for effective infectious disease management in the near and more distant future. It is too early to capture these new insights since these will keep on changing till the entire international community has recalibrated its position towards the prevention of international spread of infectious diseases in the light of a highly globalized world, and the subsequent roles for POEs.

What remains, however, is the need for a well prepared workforce at POEs both on the individual and the collective level to face COVID-19, other conventional infectious diseases such as influenza, tuberculosis, and measles, as well as any new Disease X. This geographically spread and divided workforce with widely varying needs will be one of the key players in restarting international travel and trade again. Focus on essential roles and tasks and a collaborative policy will be of utmost importance in the coming months of European COVID-19 recovery. Our findings draw attention to this crucial resource and provides a starting point for this collective approach, which, however, needs to be combined with the emerging needs of our fast-changing international society.

### Strengths and limitations

This study had several challenges that might have influenced our results. We assessed training needs on core capacity level based on the IHR [6]. Our results point out the capacities that require further attention. However, for developing training goals, these should be translated into trainable knowledge, skills, and attitudes, or so-called competencies [36, 37]. Second, we had to apply indirect sampling methods for the questionnaire since there is currently no contact network of European PoE. In this way, we had only to some degree an idea who represented the POEs in our sample and to what extent they were representative for the POE situation. Still, the ones who selected the respondents for us were partners in a European joint action program. In this way, they are reliable in selecting representatives of points of entry. Third, the low response rate from ground-crossings also is a limitation. That is why we included their response in the general results, but were not able to specifically report on ground-crossings.

Overall, a better insight into the designated POEs and the public health networks at designated POEs should remain the focus of future research to reach this group of professionals for the dissemination of new information, the invitation for education or training, and for future assessments of training needs. A more broadly distributed survey to numerous workers at each PoE would contribute to richer data than the representation of the entire POE as is the case in this study. However, this would require that personnel at POEs can be directly contacted. Last but not least, the current COVID-19 crisis has put major focus on infectious disease mangement at POEs. Our results are from a pre-COVID-19 status, in which the respondents had not perceived the crisis that they have now, but indicate with which training needs they entered the COVID-19 crisis.

## Conclusions

To our knowledge, this study is the first in Europe to assess the collective training needs of POEs from a POE perspective. In light of the current COVID outbreak, it is shown how important a prepared workforce at POE is. This study can be used during the development of the training agenda for training and exercises in the near future. We showed the issues requiring highest attention according to our sample from 50 different POEs, but above all, identified that preparedness at POEs requires a major place in European capacity building to be collaboratively ready to deal with the current and the next crisis.

## Supplementary Information

**Additional file 1.** Questionnaire Ports.pdf. The questionnaire for participants representing ports.

**Additional file 2.** Questionaire Airports.pdf. The questionnaire for participants representing airports.

**Additional file 3.** Questionnaire Ground-crossings.pdf. The questionnaire for participants representing ground-crossings.

**Additional file 4.** Additional questions on experience.pdf. Additional questions disseminated in an additional data collection round.

**Additional file 5.** Rough and prepared data.xls. In this file, the rough data file and prepared data set is presented. The file contains several sheets: Introduction: here the other sheets are introduced again. Rough File: The data as exported from Formdesk, with the headings used in Formdesk. In this sheet are highlighted: the unfinished questionnaires (orange), and questionnaires without consent for processing the data (red). Prepared: In this slide the unfinished questionnaires and those without formal consent have been excluded. Headings were added, and nominal data were splitted in colomns with binary data. Later on, the additional information from the follow-up questionnaire was added in column GL-GP. Prepared-final: Here, we excluded double submissions and non-designated points of entry. This is the database as it was uploaded into SPSS for further analysis.

**Additional file 6. **Importance & Training needs per POE type.pdf. Results for subtopics presented for ports, airports and ground-crossings seperately, including *p*-values as a result of a one-way anova analysis.

**Additional file 7.** Preferred training methodologies.pdf. A table with frequencies of preferred training methodologies presented per topic (A-H), presented for all POEs together and for respondents for ports, airports and ground-crossings seperately.

## Data Availability

All data generated or analysed during this study are included in this published article and the Additional files.

## Abbreviations

EU: European Union
IHR: International Health Regulations
JEE: Joint external evaluation
PHEC: Public health emergency contingency
PHEIC: Public health emergency of international concern
POE: Point of entry
WHO: World Health Organization
