# Supplementary material for: Assessing training needs in infectious disease management at major ports, airports and ground-crossings in Europe
Source: BMC Public Health. 2021 May 29;21:1013. doi: 10.1186/s12889-021-11008-z (PMC8164056; doi:10.1186/s12889-021-11008-z)
Supplement: Supplementary file 1 — Additional file 1. Questionnaire Ports.pdf. The questionnaire for participants representing ports. [file 12889_2021_11008_MOESM1_ESM.pdf]

Training needs assessment for designated ports (PDF of online survey)

Training Needs Assessment: Core capacities at designated ports

Pagina 1

Introduction:

Dear Sir/Madam,

We hereby present a questionnaire aimed at people responsible for or (partly) involved in preparedness and response regarding cross-border health threats, at designated ports in Europe.

Background
This training needs assessment is part of the Healty Gateways Joint Action 2018, which aims to improve preparedness and response regarding cross border health threats, and enhanced implementation of the IHR core capacities for designated points of entry in Europe. Part of this Joint Action is the development of a training program.

Goal
The goal of this questionnaire is to assess the training needs of people involved in hygiene and infectious disease prevention and control at designated ports. With the results of this assessment, we aim to set up relevant and effective training sessions.

Who are we looking for?
We are looking for people at the operational and management level at designated ports, who are involved in a.o.:
- inspection of conveyances;
- disinfection/decontamination of cargo;
- quarantine or assessment of ill travellers;
- preparedness planning, training;
- and/or crisis management work.

It might be possible that these tasks belong to several professions at your port. Please do not hesitate to consult any collegues during completion of the questionnaire for your designated port.

If you have any questions, please send an e-mail to lcionderzoek@rivm.nl

Completing the questionnaire will take about **20 minutes**. Please complete the questionnaire before **9 November 2018**.

Thank you

Terug Verder Naar overzicht formulieren Hulp vr

Completing the questionnaire will take about **20 minutes**. Please complete the questionnaire before **9 November 2018**.

Thank you.

This online questionnaire was funded by the European Union's Health Programme (2014-2020).

The content of this online questionnaire represents the views of the author only and is his/her sole responsibility; it cannot be considered to reflect the views of the European Commission and/or the Consumers, Health, Agriculture and Food Executive Agency (CHAFEA) or any other body of the European Union. The European Commission and the Agency do not accept any responsibility for use that may be made of the information it contains.

Healthy GateWays

Page 2

Privacy and personal data protection statement
This questionnaire is developed as part of the Joint Action Healthy Gateways. The data will be analyzed by the National Institute for Public Health and the Environment (RIVM) in the Netherlands (Bilthoven) (contact e-mail: LClonderzoek@rivm.nl). The questions regarding chemical threats will be analyzed by Public Health England. In accordance with the EU General Data Protection Regulation we ask your consent for the use of your data:
- The purpose of the information collected in this form is to assess training needs and to organize trainings on core capacity requirements for points of entry.
- The data can be used for (scientific) publications. It will not be further utilized for any incompatible purpose(s) and will not be passed to any third parties.
- Your contact details will only be used for communication with you, e.g. for updates and additional questions on the subject. Your contact details will be removed and erased as soon as this communication is not opportune anymore or on your first request. Your data will not be used for automated decision making. The data will be stored for 10 years.
Consent is required to process your data.

By ticking the box below, I agree and give my consent to the processing of my personal information including on this form, according to the above statement. *

☐

Please be informed that you have the right to access, correct, delete, restrict your data processing, and submit a complaint to the Data Protection Authority in accordance with Articles 15-22 of the General Data Protection Regulation (GDPR). Deleting, correcting or restriction of your data will not affect the processing of the data before its withdrawal. In order to exercise your rights you can contact us by email at the email address: LClonderzoek@rivm.nl.

Pagina 3

Personal details - Please provide the following information:

Gender: *	Make a choice
Country: *	Make a choice
Name of the designated port: *	
The sector you work in: *	Make a choice
The level you work at: *	Make a choice
Highest completed education: *	Make a choice
Job title: *	
Years of experience in your current job: *	
Do you have mangement tasks regarding the designated port?<br>If yes, how much of your working time do you spend on these tasks? *	Make a choice
Do you have operational tasks regarding the designated port?<br>If yes, how much of your working time do you spend on these tasks? *	Make a choice

Pagina 4

Instruction:
The questionnaire is divided into three parts. For each part, you will be asked to do the following:

- Please rate how important you think a topic is regarding your designated port.
- Please rate your training need for each topic.
- Please select your preference for a type of training.

Overview:
The questionnaire is divided into three parts.

First, services that could be available at all times are being assessed. Topics are:

- health risks at designated ports
- a safe environment for travellers
- routine inspection of ships
- ill travellers

Second, capacities that should be available in case response (a PHEIC)* is needed. Topics are:

- the public health emergency plan
- recommended measures
- affected persons
- affected animals

Third, a section follows about chemical hazards at ports

* during events that may constitute a public health emergency of international concern (PHEIC): an event that consitutes a public health risk to other countries through the international spread of disease and which may require an coordinated international response.

Pagina 5

The following questions regard core capacities for designated ports, which should be available ***at all times****.*
Please rate how important these topics are in your current job at the designated port. And please rate the training need you feel on these topics.

**A. Health risks at the designated port**	**Importance of the topic**					**Training Needs**				
	High	Moderate	Low	No	I don't know	Intensive	Moderate	Low	No	I don't know
1. Knowledge of public health risks from biological agents *										
2. Knowledge of public health risks from chemical agents *										
3. Knowledge of public health risks from radiological agents *										
4. Other, please specify below										

Please specify any specific training need on these topics.

Did you receive training on this topic in the last 3 years? * ○ Yes ○ No ○ I don't know

What type of training has your preference regarding this topic? *

- ☐ Case-studies
- ☐ E-module
- ☐ Presentations
- ☐ Simulation
- ☐ Discussion / knowledge exchange
- ☐ I do not have preference
- ☐ Other, please specify:

Pagina 6

The following questions regard core capacities for designated ports, which should be available ***at all times****.*
Please rate how important these topics are in your current job at the designated port. And please rate the training need you feel on these topics.

**B. A safe environment for travellers using port facilities**	**Importance of the topic**					**Training Needs**				
	High	Moderate	Low	No	I don't know	Intensive	Moderate	Low	No	I don't know
1. Inspection programs *										
2. Vector control at/near the designated port *										
3. Food- and water safety *										
4. Public washrooms; solid & liquid waste management *										
5. Air quality *										
6. Other, please specify below										

Please specify any specific training need on these topics.

Did you receive training on this topic in the last 3 years? * ○ Yes ○ No ○ I don't know

What type of training has your preference regarding this topic? *

- ☐ Case-studies
- ☐ E-module
- ☐ Presentations
- ☐ Simulation
- ☐ Discussion / knowledge exchange
- ☐ I do not have preference
- ☐ Other, please specify:

Pagina 7

*The following questions regard core capacities for designated ports, which should be available **at all times**.*
Please rate how important these topics are in your current job at the designated port. And please rate the training need you feel on these topics.

C. Routine inspection of ships	Importance of the topic					Training Needs				
	High	Moderate	Low	No	I don't know	Intensive	Moderate	Low	No	I don't know
1. Following standard operating procedures *	○	○	○	○	○	○	○	○	○	○
2. Sewage; solid- and medical wastes *	○	○	○	○	○	○	○	○	○	○
3. Food- and water safety *	○	○	○	○	○	○	○	○	○	○
4. Assessment of required health documents *	○	○	○	○	○	○	○	○	○	○
5. Ballast water management *	○	○	○	○	○	○	○	○	○	○
6. Other, please specify below	○	○	○	○	○	○	○	○	○	○

Please specify any specific training need on these topics.

Did you receive training on this topic in the last 3 years? *
○ Yes ○ No ○ I don't know

What type of training has your preference regarding this topic? *
- ☐ Case-studies
- ☐ E-module
- ☐ Presentations
- ☐ Simulation
- ☐ Discussion / knowledge exchange
- ☐ I do not have preference
- ☐ Other, please specify:

*The following questions regard core capacities for designated ports, which should be available **at all times**.*
Please rate how important these topics are in your current job at the designated port. And please rate the training need you feel on these topics.

D. Prompt assessment of ill travelers	Importance of the topic					Training Needs				
	High	Moderate	Low	No	I don't know	Intensive	Moderate	Low	No	I don't know
1. Use of protective equipment (a.o. correct type; donning and doffing) *	○	○	○	○	○	○	○	○	○	○
2. Safe removal of travellers for assessment, care, quarantine or isolation *	○	○	○	○	○	○	○	○	○	○
3. Triage (a.o. standard procedures, recognition of disease symptoms of possible contagious diseases) *	○	○	○	○	○	○	○	○	○	○
4. Identifying and calling in appropriate diagnostic facilities and medical services *	○	○	○	○	○	○	○	○	○	○
5. Other, please specify below	○	○	○	○	○	○	○	○	○	○

Please specify any specific training need on these topics.

Did you receive training on this topic in the last 3 years? *
○ Yes ○ No ○ I don't know

What type of training has your preference regarding this topic? *
- ☐ Case-studies
- ☐ E-module
- ☐ Presentations
- ☐ Simulation
- ☐ Discussion / knowledge exchange
- ☐ I do not have preference
- ☐ Other, please specify:

Instruction:

For the following questions those **during response (a PHEIC)*** will be assessed. You will again be asked to do the following:

- to assess the importance of these topics in your current job regarding the designated port.
- to rate your training needs per topic.

Thank you.

* a public health emergencies of international concern: an event that consitutes a public health risk to other countries through the international spread of disease and which may require an coordinated international response.

The following questions regard core capacities for designated ports, which should be available during ***response to events that may constitute a public health event of international concern****.*
Please rate how important these topics are in your current job at the designated port. And please rate the training need you feel on these topics.

**E. The public health emergency contingency plan**	**Importance of the topic**					**Training Needs**				
	High	Moderate	Low	No	I don't know	Intensive	Moderate	Low	No	I don't know
1. Composing and updating a public health emergency contingency plan *	○	○	○	○	○	○	○	○	○	○
2. Adequate and timely usage of the public health emergency contingency plan *	○	○	○	○	○	○	○	○	○	○
3. Arrangements between the designated port and local medical services (for a.o. isolation or treatment of ill travelers) *	○	○	○	○	○	○	○	○	○	○
4. Other, please specify below	○	○	○	○	○	○	○	○	○	○

Please specify any specific training need on these topics.

Did you receive training on this topic in the last 3 years? *

○ Yes ○ No ○ I don't know

What type of training has your preference regarding this topic? *

- ☐ Case-studies
- ☐ E-module
- ☐ Presentations
- ☐ Simulation
- ☐ Discussion / knowledge exchange
- ☐ I do not have preference
- ☐ Other, please specify:

The following questions regard core capacities for designated ports, which should be available during ***response to events that may constitute a public health event of international concern****.*
Please rate how important these topics are in your current job at the designated port. And please rate the training need you feel on these topics.

F. Recommended Measures	Importance of the topic					Training Needs				
	High	Moderate	Low	No	I don't know	Intensive	Moderate	Low	No	I don't know
1. Measures (disinsection, deratting, disinfection, decontamination) *	○	○	○	○	○	○	○	○	○	○
2. Treating goods, baggage, cargo or postal parcels *	○	○	○	○	○	○	○	○	○	○
3. Treating containers or ships *	○	○	○	○	○	○	○	○	○	○
4. Other, please specify below	○	○	○	○	○	○	○	○	○	○

Please specify any specific training need on these topics.

Did you receive training on this topic in the last 3 years? *
○ Yes ○ No ○ I don't know

What type of training has your preference regarding this topic? *
- ☐ Case-studies
- ☐ E-module
- ☐ Presentations
- ☐ Simulation
- ☐ Discussion / knowledge exchange
- ☐ I do not have preference
- ☐ Other, please specify:

The following questions regard core capacities for designated ports, which should be available during ***response to events that may constitute a public health event of international concern****.*
Please rate how important these topics are in your current job at the designated port. And please rate the training need you feel on these topics.

G. Affected persons	Importance of the topic					Training Needs				
	High	Moderate	Low	No	I don't know	Intensive	Moderate	Low	No	I don't know
1. Use of protective equipment (a.o. correct type; donning and doffing) *	○	○	○	○	○	○	○	○	○	○
2. Identification and safe use of an appropriate space for assessment, care, quarantine or isolation of affected persons *	○	○	○	○	○	○	○	○	○	○
3. Interview and triage (a.o. standard procedures, recognition of disease symptoms of possible contagious diseases) *	○	○	○	○	○	○	○	○	○	○
4. Safe transfer of suspected travellers *	○	○	○	○	○	○	○	○	○	○
5. Other, please specify below	○	○	○	○	○	○	○	○	○	○

Please specify any specific training need on these topics.

Did you receive training on this topic in the last 3 years? *
○ Yes ○ No ○ I don't know

What type of training has your preference regarding this topic? *
- ☐ Case-studies
- ☐ E-module
- ☐ Presentations
- ☐ Simulation
- ☐ Discussion / knowledge exchange
- ☐ I do not have preference
- ☐ Other, please specify:

The following questions regard core capacities for designated ports, which should be available during ***response to events that may constitute a public health event of international concern****.*
Please rate how important these topics are in your current job at the designated port. And please rate the training need you feel on these topics.

**H. Affected animals**	**Importance of the topic**					**Training Needs**				
	High	Moderate	Low	No	I don't know	Intensive	Moderate	Low	No	I don't know
1. Arrangements between the port and local veterinary services *	○	○	○	○	○	○	○	○	○	○
2. Infection control *	○	○	○	○	○	○	○	○	○	○
3. Care or treatment *	○	○	○	○	○	○	○	○	○	○
4. Other, please specify below	○	○	○	○	○	○	○	○	○	○

Please specify any specific training need on these topics.

Did you receive training on this topic in the last 3 years? *
○ Yes ○ No ○ I don't know

What type of training has your preference regarding this topic? *
- ☐ Case-studies
- ☐ E-module
- ☐ Presentations
- ☐ Simulation
- ☐ Discussion / knowledge exchange
- ☐ I do not have preference
- ☐ Other, please specify:

Instruction:

For the following questions, **chemical hazards** will be assessed. You will again be asked to do the following:

- to assess the importance of these topics in your current job regarding the designated port.
- to rate your training needs per topic.
- to select topics which you previously received training on, and the delivered and preferred type of training is asked for

Thank you.

The following questions regard core capacities for designated ports regarding ***chemical threats.***
Please rate how important these topics are in your current job at the designated port. And please rate the training need you feel on these topics.

**I. Chemical hazards** (page 1/2)	**Importance of the topic**					**Training Needs**				
	High	Moderate	Low	No	I don't know	Intensive	Moderate	Low	No	I don't know
1. Response plan to a chemical incident										
2. Notification of the appropriate individuals/authorities of a chemical incident/exposure										
3. Risks posed by chemical hazards										
4. Initial response if chemical incident/exposure is suspected										
5. Most common chemicals which pass through your port.										
6. Use of chemical personal protective equipment										
7. Apply recommended measures to isolate or decontaminate items (e.g. baggage or equipment)										
8. Apply recommended measures to transfer travellers who may be contaminated										
9. Identification of a potential chemical incident (smell, appearance, other signs)										
10. Communication with neighbouring country/ies in the event of a cross-border chemical incident										
11. Training together with other ports or points of entries										
12. Training together with neighbouring country/ies										
13. Other, please specify below										

I. Chemical hazards (page 2/2)

The following questions regard the same topics as the previous page, but cover your **experience and preference in training** on these topics.

**Did you receive training on these topics?**	Yes	No	I don't know	Delivered type of training	Preferred type of training
1. Response plan to a chemical incident				<Make a choice>	<Make a choice>
2. Notification of the appropriate individuals/authorities of a chemical incident/exposure				<Make a choice>	<Make a choice>
3. Risks posed by chemical hazards				<Make a choice>	<Make a choice>
4. Initial response if chemical incident/exposure is suspected				<Make a choice>	<Make a choice>
5. Most common chemicals which pass through your port.				<Make a choice>	<Make a choice>
6. Use of chemical personal protective equipment				<Make a choice>	<Make a choice>
7. Apply recommended measures to isolate or decontaminate items (e.g. baggage or equipment)				<Make a choice>	<Make a choice>
8. Apply recommended measures to transfer travellers who may be contaminated				<Make a choice>	<Make a choice>
9. Identification of a potential chemical incident (smell, appearance, other signs)				<Make a choice>	<Make a choice>
10. Communication with neighbouring country/ies in the event of a cross-border chemical incident				<Make a choice>	<Make a choice>
11. Training together with other ports or points of entries				<Make a choice>	<Make a choice>
12. Training together with neighbouring country/ies				<Make a choice>	<Make a choice>

Please specify any 'other' types of training on these topics.

Page 17

You are almost at the end of this questionnaire.

Optional:
I would like to be invited for further interviews on the assessed training needs, and can be reached for this purpose via the following mail address:

Did you consult other collegues for completion of this questionnaire? If yes, please tick the box.

Consulted collegue 1

The level this collegue works at: * Make a choice ▾

Job title: *

Years of experience in current job: *

Consulted collegue 2

The level this collegue works at: Make a choice ▾

Job title:

Years of experience in current job:

Consulted collegue 3

The level this collegue works at: Make a choice ▾

Job title:

Years of experience in current job:

Page 18

If you have any additional remarks or questions regarding the IHR core capacities for points of entry, this questionnaire or our research, please write them down below.

This is the end of this questionnaire.

Thank you very much for completing it, and for helping us gaining more insight in your preferences and needs regarding training content and type.

If you have any remaining questions, remarks, or if you wish to know more about the questionnaire or our research, please send an email to lcionderzoek@rivm.nl

Please do not forget to click the 'send' button to submit the questionnaire.
