# Supplementary material for: Assessing training needs in infectious disease management at major ports, airports and ground-crossings in Europe
Source: BMC Public Health. 2021 May 29;21:1013. doi: 10.1186/s12889-021-11008-z (PMC8164056; doi:10.1186/s12889-021-11008-z)
Supplement: Supplementary file 4 — Additional file 4. Additional questions on experience.pdf. Additional questions disseminated in an additional data collection round. [file 12889_2021_11008_MOESM4_ESM.pdf]

Additional file 4 – Additional questions on experience.pdf

An additional data collection round took place in July and August 2019. Below we present the body of the data collection form that was used. In addition to this form, we added the privacy statement as used in the previous questionnaire.

Data collection form:

The name of the airport / port / ground-crossing I am answering these questions for is:
………………………………………………

Response

- **Question: How often in the last 5 years did you and your colleagues need to respond to an infectious disease event at the designated point of entry you work for?**
  Answer: In the last five years, we had to respond ……………….. times.

- **Question: How many of these events took place in 2018?**
  Answer: In 2018, we had to respond ……………… times.

Exercises

- **Question: Do you have a regular schedule for multi-disciplinary exercises on infectious disease control at the point of entry?**
  Answer: [yes/no]………………

- **Question: How often in the last 5 years a multi-disciplinary exercise on infectious disease control at your point of entry was conducted?**
  Answer: In the last five years, ……………….. multi-disciplinary exercises took place.

- **Questions: How many of these exercises took place in 2018?**
  Answer: In 2018, ………………… multi-disciplinary exercises took place.
