# Supplementary material for: Assessing training needs in infectious disease management at major ports, airports and ground-crossings in Europe
Source: BMC Public Health. 2021 May 29;21:1013. doi: 10.1186/s12889-021-11008-z (PMC8164056; doi:10.1186/s12889-021-11008-z)
Supplement: Supplementary file 6 — Additional file 6. Importance & Training needs per POE type.pdf. Results for subtopics presented for ports, airports and ground-crossings seperately, including p-values as a result of a one-way anova analysis. [file 12889_2021_11008_MOESM6_ESM.pdf]

Additional File 6 –Importance & Training needs per POE type.pdf

Mean scores on training needs and importace for subtopics, presented for ports, airports and ground-crossings seperately, including p-values as a result of a one-way anova analysis. The subtopics A1 – H3 correspond with those presented in the main article.

Legend:

Color	Mean score
(white)	<1.0
(light grey)	1≤x<1.5
(light green)	1.5≤x<2
(grey)	2≤x<2.5
(dark grey)	2.5≤x≤3
(black)	No data available

Training needs (mean)	Ports	Airports	Ground-crossings	One-way ANOVA P-Value	Importance (mean)	Ports	Airports	Ground-crossings	P-value
A1	1.85	2.06	2	0.698	A1	2.37	2.61	2.2	0.45
A2	1.85	1.76	1.8	0.948	A2	1.96	2.06	2	0.93
A3	1.62	1.63	2	0.623	A3	1.65	1.82	2.2	0.392
B1	2.04	1.76	2	0.675	B1	2.56	2.18	2.2	0.282
B2	2.11	1.78	2	0.544	B2	2.44	2	2.2	0.306
B3	2.07	1.44	2	0.138	B3	2.59	2.11	2.2	0.21
B4	1.81	1.33	0.8	0.072	B4	2.3	1.89	1.4	0.058
B5	1.64	1.38	0.8	0.203	B5	1.96	1.61	0.8	0.053
C1	2.11	1.94	2	0.856	C1	2.56	2.35	2.4	0.705
C2	2	1.47	1.2	0.091	C2	2.19	1.94	1.8	0.608
C3	2.07	1.59	1.8	0.332	C3	2.48	2	2.2	0.266
C4	2.04	1.65	2	0.519	C4	2.48	2.12	2	0.365
C5	1.92				C5	1.92			
D1	2.21	2.35	2	0.753	D1	2.44	2.67	2.2	0.479
D2	2.25	2.24	1.8	0.629	D2	2.4	2.56	2	0.476
D3	2.21	2.29	1.6	0.405	D3	2.4	2.67	1.8	0.201
D4	2.08	2.06	1.4	0.346	D4	2.28	2.5	1.6	0.17
E1	1.93	2	1.8	0.922	E1	2.7	2.51	1.6	0.008
E2	2	2.18	1.6	0.515	E2	2.56	2.61	1.6	0.027
E3	2.04	1.76	1.25	0.302	E3	2.56	2.5	1.25	0.018
F1	2.27	2.06	2	0.699	F1	2.62	2.71	2.2	0.307
F2	2.15	1.94	1.4	0.319	F2	2.31	2.18	2	0.747
F3	2.15	2.24	1.2	0.07	F3	2.38	2.35	1.8	0.273
G1	2.29	2.29	2.2	0.987	G1	2.69	2.72	2.4	0.621
G2	2.25	2.18	1.8	0.608	G2	2.58	2.67	2	0.198
G3	2.08	2.06	1.8	0.853	G3	2.56	2.61	1.8	0.114
G4	2.04	2.06	1.8	0.864	G4	2.52	2.44	1.8	0.201
H1	1.57	1.5	0.8	0.345	H1	1.75	1.82	1.2	0.516
H2	1.62	1.56	0.6	0.182	H2	1.91	2	0.8	0.097
H3	1.48	1.31	0.6	0.294	H3	1.74	1.71	0.8	0.193
