# Supplementary material for: Assessing training needs in infectious disease management at major ports, airports and ground-crossings in Europe
Source: BMC Public Health. 2021 May 29;21:1013. doi: 10.1186/s12889-021-11008-z (PMC8164056; doi:10.1186/s12889-021-11008-z)
Supplement: Supplementary file 7 — Additional file 7. Preferred training methodologies.pdf. A table with frequencies of preferred training methodologies presented per topic (A-H), presented for all POEs together and for respondents for ports, airports and ground-crossings seperately. [file 12889_2021_11008_MOESM7_ESM.pdf]

Additional File 7 – preferred training methodologies

A table with frequencies of preferred training methodologies presented per topic (A-H), presented for all POEs together and for respondents for ports, airports and ground-crossings seperately. Highest scores per topic are marked in green. Highest scores per methodology are scored in blue, and where these overlap it is marked yellow. Per score, the absolute number of votes per POE type are shown as (ports, airports, ground-crossings).

Topic	Case Study	E-module	Presentation	Simulation	Discussion	No preference	Other
**A. Different health risks**	23 (15,6,2)	27 (16,9,2)	33 (18,12,3)	28 (15,10,3)	27 (14,12,1)	4 (3,1,0)	2 (2,0,0)
**B. Safe Environment**	23 (15,6,2)	26 (17,7,2)	31 (18,11,2)	22 (13,7,2)	29 (17,11,1)	6 (3,2,1)	2 (2,0,0)
**C. Routine inspections**	23 (15,7,1)	27 (19,6,2)	31 (17,11,2)	19 (12,6,1)	24 (12,10,2)	6 (3,2,1)	1 (1,0,0)
**D. Assessment of ill travelers**	20 (11,6,3)	24 (15,7,2)	25 (15,9,1)	28 (14,11,3)	25 (12,11,2)	6 (4,1,1)	4 (3,1,0)
**E. PHEC plan**	20 (12,6,2)	22 (14,6,2)	27 (14,11,2)	23 (11,10,2)	26 (10,14,2)	6 (4,1,1)	1 (1,0,0)
**F. Recommended measures**	21 (13,6,2)	26 (16,8,2)	31 (17,11,3)	23 (12,9,2)	26 (14,11,1)	6 (3,2,1)	1 (1,0,0)
**G. Exposed/ sick persons**	21 (12,6,3)	24 (13,9,2)	27 (14,11,2)	27 (14,10,3)	24 (12,10,2)	7 (5,1,1)	2 (1,1,0)
**H. Affected animals**	13 (10,2,1)	19 (11,7,1)	22 (13,8,1)	15 (10,4,1)	18 (9,9,0)	14 (8,4,2)	1 (1,0,0)
**Total score**	**164 (103,45,16)**	**195 (121,59,15)**	**227 (126,84,16)**	**185 (101,67,18)**	**199 (100,88,11)**	**55 (33,14,8)**	**14 (12,2,0)**

PHEC = public health emergency contingency
